# Ruptured Distal Superior Cerebellar Artery Dissecting Aneurysm Treated with a Flow-diverting Device: Case Report and Review of Literature

**DOI:** 10.7759/cureus.2918

**Published:** 2018-07-03

**Authors:** Rizwan Tahir, Karam P Asmaro, Sameah Haider, Max Kole

**Affiliations:** 1 Neurological Surgery, Henry Ford Hospital, Detroit, USA; 2 Neurosurgery, Henry Ford Health System, Detroit, USA; 3 Neurosurgery, Henry Ford Hospital, detroit, USA

**Keywords:** aneurysm, flow-diverting device, fusiform aneurysm, superior cerebellar artery

## Abstract

Distal fusiform aneurysms of the superior cerebellar artery (SCA) are rare and present several challenges to clinicians, especially when ruptured. While several treatment options are available, including surgical clipping and endovascular coiling, numerous challenges still remain due to the presence of vital neighboring neurovascular structures. In addition, the complications that arise due to the compromise of brainstem perforators make these aneurysms difficult to treat.

This case report demonstrates the successful treatment of a ruptured fusiform aneurysm of the SCA with a flow-diverting device. We also conducted a literature review of the use of flow-diverting devices for treating such aneurysms.

When choosing a treatment modality for a ruptured aneurysm, clinicians must consider both the patient-specific variables as well as aneurysm morphology. Treatment options including microsurgical clipping, endovascular coiling, and flow diversion carry risks. Therefore, the clinician must decide which option best fits each situation.

## Introduction

Superior cerebellar artery (SCA) aneurysms are rare, with a reported incidence of 0.2% [1] and fusiform aneurysms of the SCA are still rarer. Treatment includes surgical approaches and endovascular techniques. We present a case of a ruptured distal dissecting SCA aneurysm in a young female, which we treated with a flow-diverting device (FDD) to preserve the flow through the parent vessel. 

## Case presentation

History & examination

A 31-year-old African-American female with no significant medical history presented with a sudden onset of severe headaches that were highly suggestive of subarachnoid hemorrhage (SAH). The patient was initially evaluated in the emergency department where a neurological examination revealed no deficits.

Pathological findings

**Figure 1:**
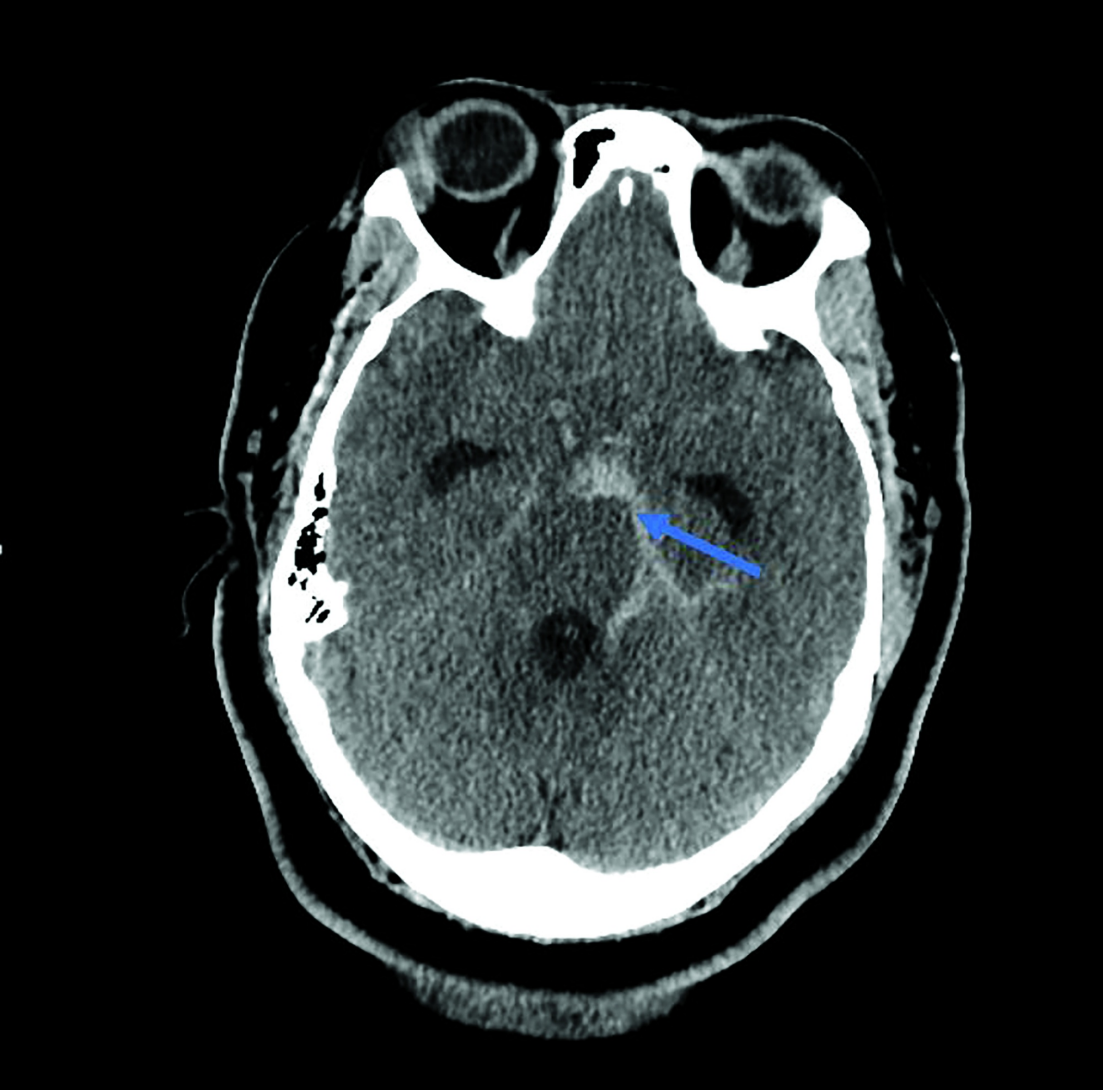
Computed tomography of the head Computed tomography (CT) of the head demonstrating asymmetric subarachnoid hemorrhage in the left ambient and quadrigeminal cisterns (blue arrow) with early hydrocephalus.

A computed tomography (CT) scan revealed a SAH filling the pre-pontine, crural, ambient, and quadrigeminal cisterns more prominently on the left side of the brain, with mild hydrocephalus (Figure 1). A CT-angiography (CTA) showed an enlarged vessel within the left ambient cistern. Digital subtraction angiography (DSA) also revealed the large and irregular appearance of the left SCA in the ambient cistern. No immediate intervention was undertaken.

**Figure 2:**
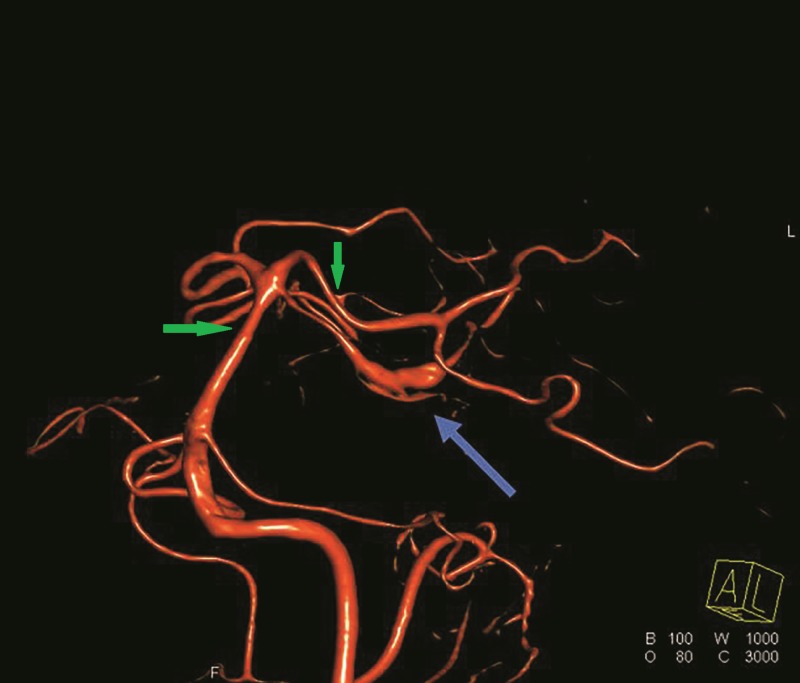
Three-dimensional reformatted digital subtraction angiography Three-dimensional reformatted digital subtraction angiography demonstrating left fusiform aneurysm of the superior cerebellar artery (SCA) (blue arrow). Mild vasospasm can be seen in the distal basilar artery and bilateral SCA and posterior cerebellar artery (green arrows).

The patient was admitted to the neurosurgical intensive care unit (ICU) and treatment was initiated with regular neurological examinations and daily transcranial-doppler (TCD) studies. The patient remained intact, with TCD values showing only moderate vasospasm. The patient did not require any treatment for the mild radiographic hydrocephalus. On Day 14, the patient underwent a repeat DSA which showed a persistent vasospasm in the distal basilar and bilateral posterior cerebral arteries. Three-dimensional (3-D) reformatting also demonstrated a dissecting fusiform SCA aneurysm in the lateral ponto-mesencephalic segment (Figure 2). Due to the persistent vasospasm, no intervention was undertaken at that time as definitive treatment measures were being discussed in a multidisciplinary board meeting.

Endovascular intervention

On Day 21, the patient underwent a successful placement of a low-profile visualized intraluminal support (LVIS Jr.) device (MicroVention, Tustin, CA) covering the fusiform aneurysm, which measured 3-mm wide and 18-mm in length. After premedication with aspirin and clopidogrel, a 7-Fr sheath was inserted into the common femoral artery and a 6-Fr guide catheter was inserted into the left vertebral artery. The left SCA was catheterized using the microcatheter and microguidewire. The LVIS Jr. device was then deployed successfully along the fusiform segment of the SCA without evidence of contrast extravasation. The treatment goal was to preserve the intraluminal flow with flow diversion from the aneurysm for reduction and eventual obliteration.

Postoperative course

**Figure 3:**
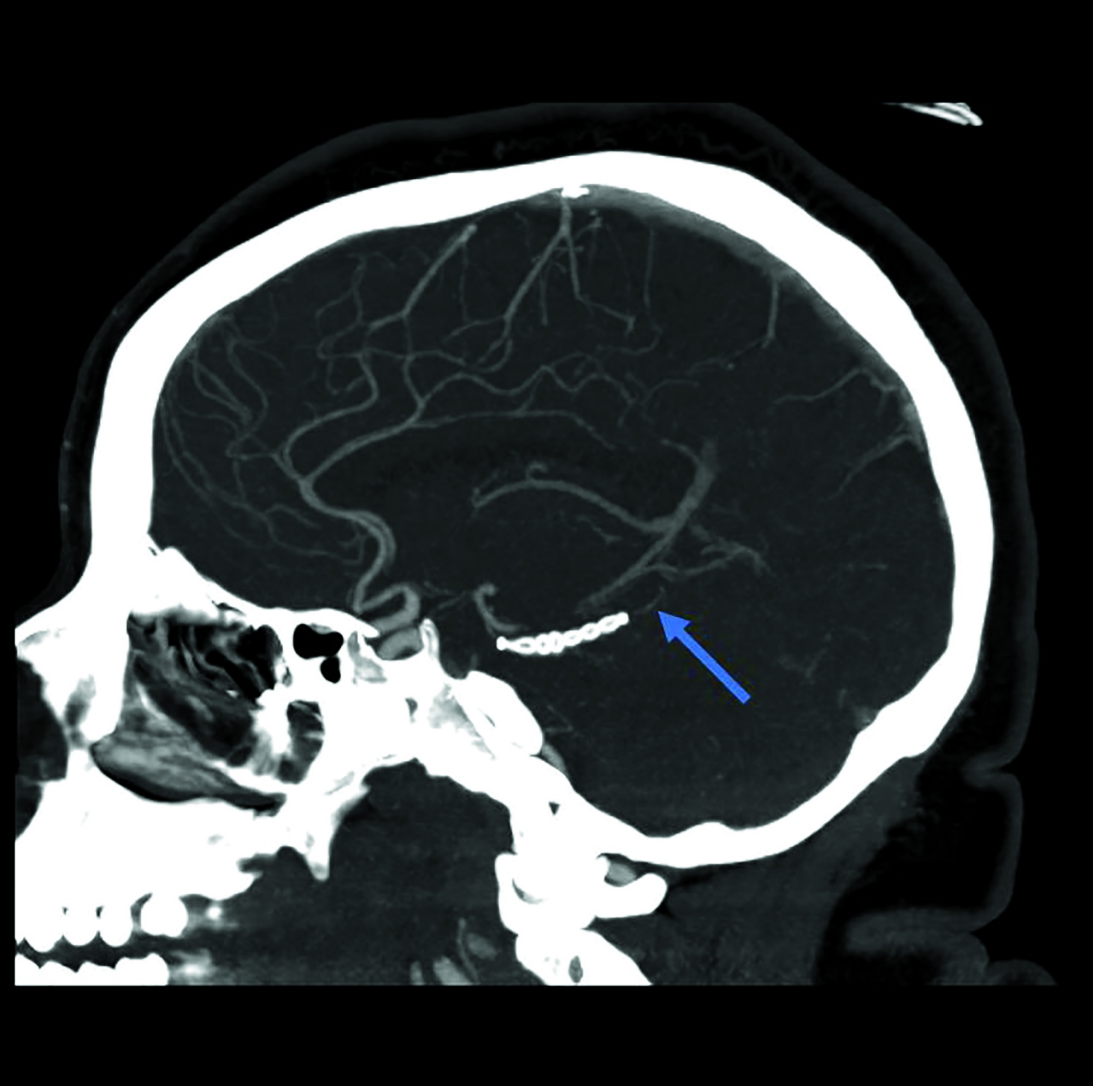
Computed tomography angiogram Computed tomography angiogram at the 3-month follow-up demonstrating the left superior cerebellar artery low-profile visualized intraluminal support Jr. stent with preserved flow both proximal and distal to stent (blue arrow).

**Figure 4:**
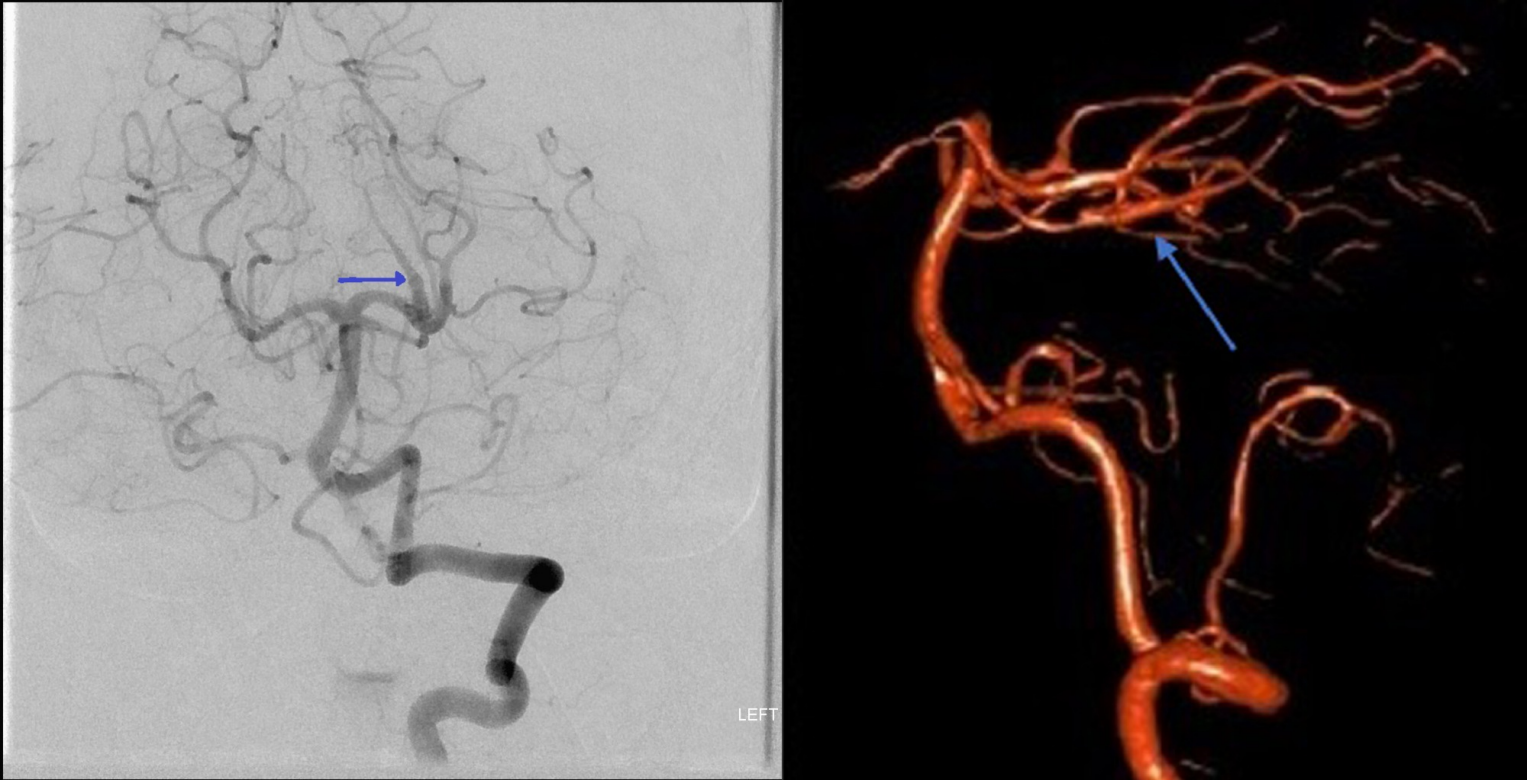
Digital subtraction angiography Digital subtraction angiography at the 10-month follow-up demonstrating the interval healing of the fusiform superior cerebellar artery (SCA) aneurysm with preserved distal vessel patency although mild distortion caused by the stent (blue arrow).

At the three-month follow-up, the patient had no complaint and was neurologically intact on examination. A CTA performed at that time showed the SCA patency proximal and distal to the stent without evidence of persistent dilatation or an aneurysm (Figure 3). A formal catheter angiogram was deferred by the patient. At the 10-month follow-up, a DSA demonstrated the interval healing of the aneurysm with preserved vessel patency as well as mild distortion caused by the stent in the SCA (Figure 4). The patient remained neurologically intact on examination with no complaints.

## Discussion

Several cases have been published regarding the management of ruptured fusiform SCA aneurysms with endovascular treatments prevailing over surgical options. Open surgical options include trapping with or without bypass, clipping of the aneurysmal body, and wrapping [2]. Endovascular treatment with parent artery occlusion with coils has been described as the most common treatment option. Complications from this procedure have ranged from cranial nerve palsies [3], diplopia [1], intra-procedural rupture [1], and death [4].

**Figure 5:**
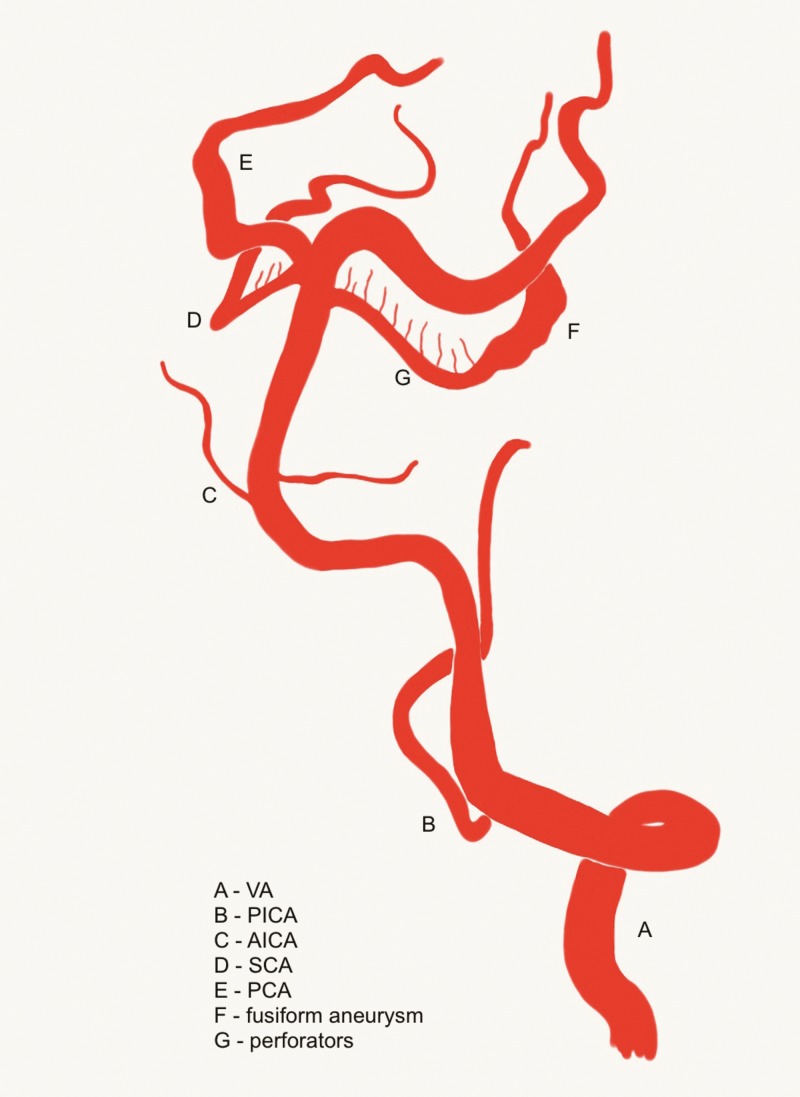
Artist's three-dimensional illustration Artist’s three-dimensional illustration of posterior circulation anatomy depicting a left-sided fusiform superior cerebellar artery (SCA) aneurysm. Brainstem perforating arteries from the SCA are shown arising proximal to the aneurysm.

The SCA contains four segments: the anterior ponto-mesencephalic, the lateral ponto-mesencephalic, the cerebello-mesencephalic, and the cerebellar cortical segment. The lateral ponto-mesencephalic segment contains perforators to the inferior colliculus and brainstem tegmentum ranging from none to as many as 10 (Figure 5) [5]. The distal SCA segments give blood supply to the superior cerebellum, the superior vermis, and the cerebellar white matter, all of which receive collaterals from the anterior inferior cerebellar artery (AICA) and the posterior inferior cerebellar artery (PICA).

In 2011, Nelson published the largest study on FDDs used for intracranial aneurysms in the pipeline embolization device for the intracranial treatment of aneurysms (PITA) [6]. FDDs were found to preserve parent vessel patency while promoting thrombosis in aneurysms adjacent to the stent by altering the flow dynamics across the wall of the device. The thrombosis was not immediate but was seen in 93% of cases in the six-month follow-up. The PITA investigators also highlighted the complications related to both the occlusion of perforators across the device and “kinking” through tortuous segments that made delivery challenging. The reported post-procedural complication rate was 6.5% [6].

The ruptured SCA aneurysm in our patient presented several challenges to treatment. Our patient was right-handed with a body mass index of 52.27. Therefore, a craniotomy via a sub-temporal approach would pose several risks including damage to brainstem perforators and a retraction injury to the dominant temporal lobe during exposure. Additionally, the fusiform morphology would not allow for a simple clipping but rather, a clip reconstruction. An endovascular approach also presented challenges. Coil occlusion would be difficult due to the fusiform morphology. And perforators supplying the midbrain would be at risk if the vessel occluded during coiling. Stenting through the parent vessel would preserve distal vessel patency but (in a ruptured aneurysm) would also require the administration of dual anti-platelet medications which could result in a devastating hemorrhage if the aneurysm re-ruptured. We felt that if an occlusion of the parent vessel occurred distal to the perforators, then a stroke could be clinically silent due to collateral supply from the AICA and PICA. Thus, the decision was made to delay treatment until angiographic evidence of the vasospasm was resolved and then to pursue treatment with a newly designed FDD – the LVIS Jr.

Wang et al. highlighted the flow-diverting properties of the LVIS Jr stent and compared them with those of the pipeline embolization device (PED) and the enterprise stent [7]. Wang concluded that the LVIS Jr was superior to the enterprise but carried less flow-diverting effects than the PED. And as Nelson described in the PITA trial, the delivery of the pipeline device was difficult in tortuous segments of the parent vessel [6]. The lateral ponto-mesencephalic segment makes a sharp bend around the crus cerebri, which would be difficult to navigate with the PED. The LVIS Jr device, however, can be deployed with a smaller microcatheter, which poses a lesser risk of dissection during delivery. Balancing these two concepts, we chose the LVIS Jr. The procedure was completed without complications and no neurological deficit was appreciated post-procedurally.

A literature review using PubMed and Google Scholar yielded one reported case of an SCA aneurysm treated with an FDD. Briganti described a ruptured proximal SCA aneurysm treated with a PED [1] which was deployed from the posterior cerebral artery to the basilar artery across the origin of the SCA. Post-procedure SCA filling was delayed and diminished but resolved on a follow-up DSA at six months, with no signs of aneurysm refilling. The patient suffered no neurological sequelae from her rupture or treatment [1].

## Conclusions

When choosing a treatment modality for a ruptured aneurysm, clinicians must consider both patient-specific factors as well as aneurysm morphology challenges. All treatment options including microsurgical clipping, endovascular coiling, and flow diversion carry risks. Therefore, the clinician must decide which option best fits each situation.
